# *Mycobacterium nanjing* sp. nov. Isolated from Cutaneous Infection, China

**DOI:** 10.3201/eid3203.252001

**Published:** 2026-03

**Authors:** Yidie Zou, Youming Mei, Wenyue Zhang, Ying Shi, Haiqin Jiang, Yukun Huang, Hongsheng Wang

**Affiliations:** Chinese Academy of Medical Sciences and Peking Union Medical College, Nanjing, China

**Keywords:** tuberculosis and other mycobacteria, *Mycobacterium*, *Mycobacterium nanjing*, skin diseases, infections, bacteria, China

## Abstract

We report a case of a cutaneous infection in an immunocompetent person in China caused by an uncharacterized *Mycobacterium* strain. The patient isolate was identified as a novel species by whole-genome sequencing. We propose *Mycobacterium nanjing* sp. nov. as the name for this new species.

Mycobacterial infections are a major public health concern and pose a continued threat to human health. The incidence and prevalence of nontuberculosis mycobacteria (NTM) infections are on the rise in certain regions and might surpass the rates of tuberculosis (*1*). In southeastern coastal China, the *Mycobacterium avium* complex, particularly *M. intracellulare*, predominates, followed by rapidly growing mycobacteria such as the *M. abscessus* complex. *M. kansasii* has been reported in some coastal cities (*2*). The relatively high NTM case count might be associated with warm climates, urban water systems, and improved laboratory detection (*3*–*5*). We report a case of cutaneous infection in Jiangsu Province, China, caused by an uncharacterized *Mycobacterium* strain, for which we propose the name *Mycobacterium nanjing* sp. nov.

In 2025, an 86-year-old man was admitted to the outpatient department with a subcutaneous nodule on his left palm. The patient reported the nodule appeared 1 month earlier after a minor penetrating trauma to the left palm caused by a wooden splinter during carpentry work. Physical examination revealed 2 red subcutaneous nodules on the left hand, 1 in the palm and 1 on the back of the hand, each measuring 0.5 cm (Figure). Laboratory tests revealed a reduced erythrocyte count of 3.82 × 10^9^ cells/L (reference range 4.3–5.8 × 10^12^ cells/L) and hemoglobin of 117 g/L (reference range 130–175 g/L). Serologic tests for syphilis, HIV, hepatitis B and C viruses, and tuberculosis were all negative. 

**Figure:**
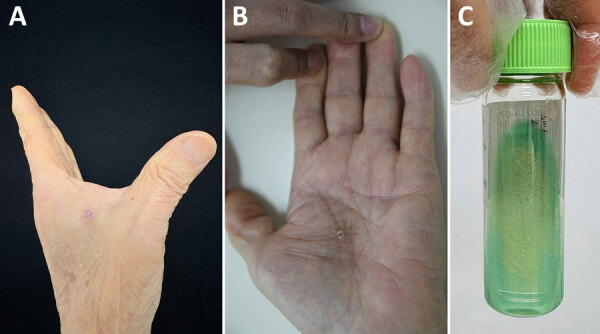
Novel cutaneous *Mycobacterium* infection in an 86-year-old man in China. A, B) Subcutaneous nodules on the left hand of the patient. C) *Mycobacterium* colonies grown on modified Löwenstein–Jensen medium slants.

Color doppler ultrasound revealed 3 hypoechoic masses in the dermis and subcutaneous fat layer of the left palm and back of hand lesions. The larger mass was in the palm, with uneven echo in the inner part, irregular shape, approximately 2.25 × 1.17 cm in size, approximately 0.93 cm in thickness, unclear boundary, no capsule, and enhanced echo in the surrounding soft tissue. Abundant blood flow was seen in and around the masses. 

Histopathologic examination revealed hyperplasia of the epidermis and lymphocyte-dominated inflammatory cell infiltration in the superficial dermis. We conducted PCR testing on skin specimens by using common mycobacterial primers and cultured the samples on Löwenstein–Jensen slants. All PCRs included a no-template negative control that remained amplification-free. Direct PCR results were inconclusive. After 15 days of incubation at 37°C, we observed light yellow colonies on modified Löwenstein–Jensen medium slants (Figure). The organism was photochromogenic with a smooth appearance and slow growth rate.

We extracted DNA from the colonies for PCR analysis and aligned the sequences by using BLAST (https://blast.ncbi.nlm.nih.gov/Blast.cgi) for species identification. The partial sequence of the *hsp65* gene (736-bp) shared greatest similarity with *Mycolicibacterium gilvum* Spyr1 (95.18%), and the *rpoB* gene (435-bp) shared greatest similarity with *Mycolicibacterium vanbaalenii* strain L1I3 (97.30%). On the basis of those results, we propose this strain might be a new member of the *Mycobacterium* genus.

For precise pathogen species identification, we subjected the patient isolate, ZZG, to whole-genome sequencing on the DNBSEQ platform (MGI, https://mgi-tech.eu) at the Beijing Genomics Institute (Shenzhen, China), yielding 1,177 Mb of data at 195× depth. Whole-genome sequencing yielded a genome assembly of 5.75 Mb (6,029,915 bp) for isolate ZZG, with an overall GC content of 67.87%. We submitted the whole genome to the type strain genome server (https://tygs.dsmz.de) to evaluate digital DNA-DNA hybridization with all available mycobacterial genomes (Table). The result suggests this strain is closest to *M. vaccae* (ATCC no. 15483). The digital DNA-DNA hybridization value is 28.9% and was calculated by using the genome-to-genome distance calculator formula, which is far from the threshold value for species delineation (70%). For phylogenetic analysis, the 5 species most closely related to isolate ZZG identified by the Type Strain Genome Server were involved; *M. marinum* (ATCC no. 927) was the outgroup. We constructed a phylogenetic tree from core genes by using RAxML (https://github.com/amkozlov/raxml-ng) under the general time-reversible plus invariable site plus discrete Gamma model with 1,000 bootstrap replicates, which demonstrated that strain ZZG was a distinct monophyletic clade, independent of its closest relatives (Appendix Figure, http://wwwnc.cdc.gov/EID/article/32/3/25-2001-App1.pdf). We propose *Mycobacterium nanjing* sp. nov. as the name for this potential new species.

**Table:** Genomic relatedness of novel strain ZZG recovered from patient with cutaneous *Mycobacterium* infection to closely related *Mycobacterium* species, China*

Subject strain	dDDH (95% CI), %†	G+C content difference
*M. vaccae* ATCC 15483	28.9 (26.5–31.4)	0.71
*M. vanbaalenii* PYR-1	28.4 (26.1–30.9)	0.08
*M. austroafricanum* DSM 44191	28.2 (25.9–30.7)	0.19
*M. parafortuitum* CCUG 20999	24.8 (22.5–27.3)	0.66
*M. gilvum* NCTC 10742	24.4 (22.1–26.9)	0.23
*M. rufum* DSM 45406	23.7 (21.4–26.1)	1.37

*dDDH, digital DNA-DNA hybridization. †Calculated using the genome-to-genome distance calculation formula.

We initially treated the patient with moxifloxacin (400 mg 1×/d), and clarithromycin (250 mg 2×/d) for 14 days. Antimicrobial drug susceptibility testing then revealed susceptibility to meropenem, linezolid, ciprofloxacin, moxifloxacin, tobramycin, minocycline, trimethoprim/sulfamethoxazole, doxycycline, amikacin, and rifabutin but resistance to rifampin and amoxicillin. We prescribed a roxithromycin regimen (150 mg 2×/d) for the patient on the basis of those results.

In summary, we isolated a distinct *Mycobacterium* species from a patient with a cutaneous infection. We propose the name *Mycobacterium nanjing* sp. nov. for this species. *M. nanjing* can cause disease in immunocompetent patients and shows susceptibility to multiple antimicrobial drugs. Despite the advantages of direct molecular detection in sensitivity and convenience, culture remains the standard and an indispensable component of a complementary diagnostic strategy, particularly for emerging mycobacterial species. The combination of surgical resolutions with antimicrobial therapy could be a good option for patients with *Mycobacterium*–caused cutaneous infections.

Appendix Additional information about *Mycobacterium nanjing* sp. nov. isolated from cutaneous infection, China
