# Supplementary material for: Mycobacterium nanjing sp. nov. Isolated from Cutaneous Infection, China
Source: Emerg Infect Dis. 2026 Mar;32(3):452–4. doi: 10.3201/eid3203.252001 (PMC13016007; doi:10.3201/eid3203.252001)
Supplement: Appendix — Additional information about Mycobacterium nanjing sp. nov. isolated from cutaneous infection, China [file 25-2001-Techapp-s1.pdf]

Article DOI: https://doi.org/10.3201/eid3203.252001

EID cannot ensure accessibility for supplementary materials supplied by authors. Readers who have difficulty accessing supplementary content should contact the authors for assistance.

Mycobacterium nanjing sp. nov. Isolated from Cutaneous Infection, China

Appendix

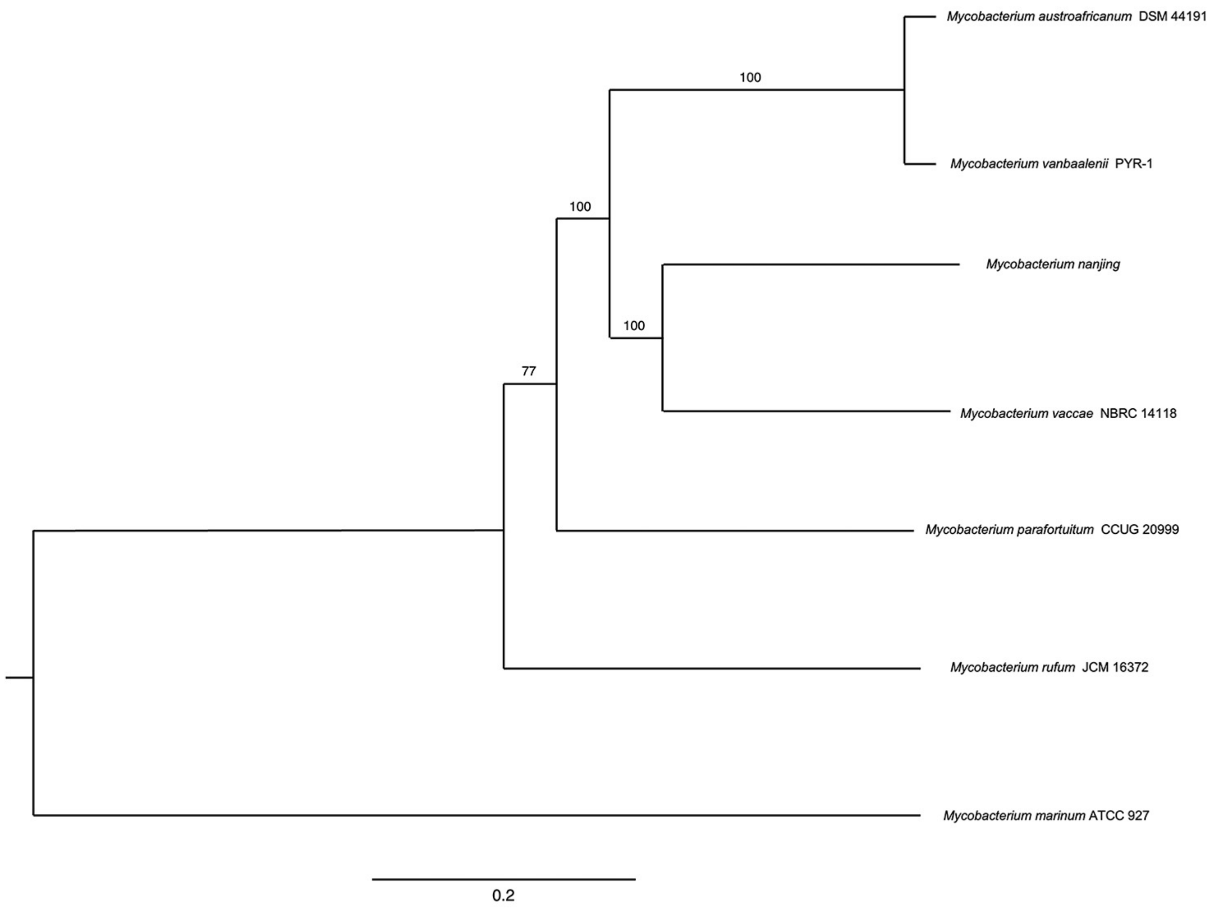

Appendix Figure. Genome sequence-based tree of *Mycobacterium nanjing* infection in an 86-year-old man in China.
